# Research on the critical technique of synchronous rotation construction with large angle for T-shape curve rigid frame bridge

**DOI:** 10.1038/s41598-022-05403-8

**Published:** 2022-01-27

**Authors:** Junhu Shao, Mengjun Duan, Wei Yang, YongZhen Li

**Affiliations:** 1 School of Architecture and Civil Engineering, Chengdu University, Chengdu, 610106 China; 2 China Railway Engineering Consulting Group Co. Ltd., Beijing, 100055 China; 3 JSTI GROUP, Nanjing, 210017 China

## Abstract

The superstructure rotation method (SRM) has been widely used in recent years due to its rapid construction, low cost and less impact on existing traffic.This paper focuses on construction method and the key parameters related with large angle synchronous rotation construction of T-shape curved rigid frame bridges, taking two bridges of the Wuyi Expressway over the Chengdu-Kunming Railway as the engineering background.The results show that the construction methods used in this project can accomplish the realization of complexed synchronous rotation execution of T-shape curve rigid frame bridge. The construction methods consist of the installation process of ball joint, the design of traction system, accuracy control method and rotation control strategy.The friction coefficients from practical measurements were compared with the analytical ones from existing formulas, and it shows that the calculation method can give good predictions for the friction coefficients at the SRM of curve rigid frame bridge.Finally, the key technologies and determination of key parameters applicable for large angle synchronous rotation construction of curve T-shape rigid frame bridge are summarized. Furthermore, the research results in this paper can provide technical recommendation for the construction of the similar bridges.

## Introduction

With the rapid development of transportation, the cross-over between traffic lines becomes more and more frequent, the bridge cross over the traffic line will impact the traffic operation of existing lines inevitably. Under the premise of reducing the impact on existing lines as much as possible, safe and fast construction has become an urgent problem to be solved. In this background, the upper structure rotation construction method has drawn great attention from engineers.

The superstructure rotation method (SRM) includes building the bridge superstructure parallel to the obstacle being crossed (cliff, rivers, or existing lines) and then rotating it into its final position. According to the different directions of rotation, it can be divided into three types: vertical rotating construction, horizontal rotating construction and a combination of the above two constructions^1–3^. This study focuses on horizontal rotating construction, which is also called the accelerated bridge construction (ABC) method, provides the optimal urban bridge construction solution by reducing the impact on traffic, improving safety, shortening the project duration, and optimizing the project budget^4,5^. It significantly simplifies and accelerates the construction of bridges, and is much more efficient than other methods that are possibly expensive, resource-wasting, and time-consuming^6^.

For the construction of urban flyover, the superstructure horizontal rotation method is widely used, the main steps of this method are shown as follows. Firstly, the pier is built on both sides of the obstacle, and then the rotating structure is installed at the top or bottom of each pier. After that, two half-span bridges are built, and each half-span is rotated to its final position by using the rotating mechanism. Finally, the structure is locked and closed after rotation^7^. The key point in this process is the design of the rotating structure, which usually adopts the spherical hinge. Additional circular rotating balls are needed in large-span bridges. The design of the structure depends on the weight, structure (mainly weight distribution), and site characteristics of each span.

The SRM method was first applied in the 1940s. After decades of development and improvement, it has been successfully applied to hundreds of bridges throughout the world, including North America, Austria, Europe, and Asian countries. This method is especially suitable for arch bridge^8,9^, cable-stayed bridge^10–13^, continuous girder bridge^14^ and so on^15–18^^.^ It was first invented as a pontoon bridge for military utilisation during World War II Statistical studies^19^ have shown that the SRM method has been widely used in various bridge types after years of development. Especially after 2000, it ushered in a qualitative leap, the continuous expansion of the rotating tonnage, and even exceeded 30,000 tons in 2018 (Tangshan Erhuan Road swivel cable-stayed bridge, 33,000 t). With the prosperity of the economy and the increase in traffic volume, this growth trend will continue in the near future.

The calculation of the unbalanced moment and the friction coefficient is the key parameter in SRM, the unbalanced moment and the counterweight of the bridge are obtained by the test of the unbalanced weight, which can affect the stability and safety of the construction process. Generally, the unbalanced moment is obtained by applying a jacking force at the spherical hinge, and this method has been already applied in SRM of straight bridges with quite good results^20,21^. The friction coefficient of the spherical hinge determines the accuracy for the prediction of the traction force, which is the crucial parameter of SRM. Huge traction force may cause the construction process more complexed and even lead to the overturning of the structure. At present, it is generally assumed that the arc-shaped friction surface is planar, and a simplified formula is derived. Based on this formula and weighing test, the friction coefficient can be predicted^22,23^.

The existing researches have studied the key problems of the SRM method, but there is no detailed description of the SRM construction process, including the construction steps and schemes, the stress monitoring schemes of the spherical hinge. The existing research focuses on SRM of single straight bridges, and the synchronous rotation construction with large angles of curve bridge research has not yet involved. For the synchronous rotation construction with large angles of the curved bridge, the synchronization of the double bridge at the construction process should be considered, which ensures that the bridge does not collide during construction. However, there is no report on the control strategy of the synchronous rotation construction with large angles of the curved bridge. In addition, the unbalanced weight of curved bridge at longitudinal and transverse will affect the friction coefficient of the ball joint in the construction process, this special state will affect the friction coefficient of the spherical hinge in the construction process. The friction coefficient prediction formula in the existing literature is derived base on the straight bridge, thus further research needs to be carried out if we want to apply this method for the curved bridge with SRM. Therefore, the conventional prediction method of friction coefficient may lead to inaccurate prediction of traction force, which makes bridge superstructure difficult to rotate. Besides, the bridge superstructure rotation distance cannot be predicted accurately when construction is completed, which makes the bridge difficult to closure.

Hence, this study focuses on the construction process of two bridges with one synchronous rotation construction and presents the key technologies in the rotation construction method, including the installation of the spherical hinge, the design of the traction system, and the control scheme of each main structure. The theoretical parameters in the rotation process are also calculated in detail, especially the static friction factor of the spherical hinge is theoretically deduced and corrected. By comparing with the actual engineering test results, the error of different friction coefficient prediction methods is evaluated. It is hoped that this study can provide a reference for subsequent engineering practice and provide inspiration for other designers.

## Bridge description

The bridges selected for this paper are two T-shaped rigid frame bridges of the Wuyi Expressway, China. These two T-shaped rigid frame bridges cross the existing Chengdu-Kunming Railway which is one of the main-line railways in Southwest China and one of China's three horizontal and five vertical main-line railway networks. Due to its tremendous amount of traffic, building bridges on it should minimize the impact on the traffic and shorten the construction period as much as possible. So the SRM method was selected eventually.

**Figure 1:**
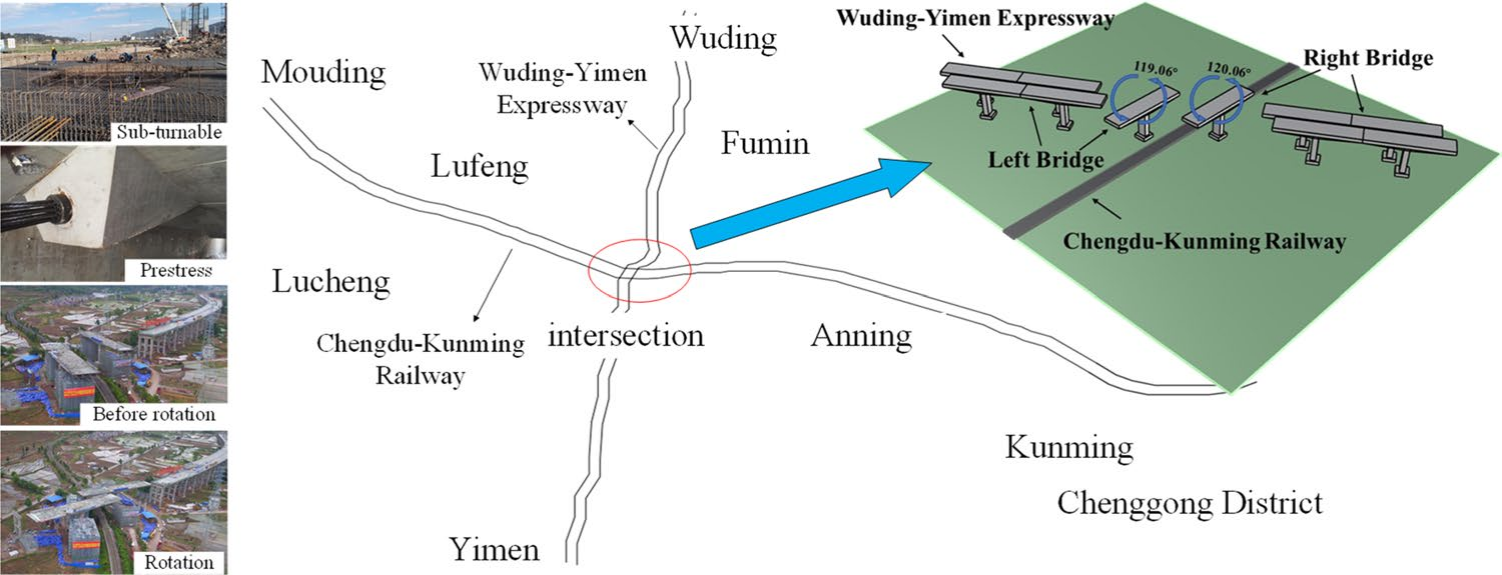
Layout of synchronous rotation.

Additional geometric details for the bridges are given in Table 1. The relative position of the swivel bridge and the Chengdu-Kunming highway is showed in Fig. 1.

**Table 1:** Geometric specification of two bridges.

	Left bridge	Right bridge
Bridge type	T-shape rigid frame bridge	T-shape rigid frame bridge
Girder type	Box girder (one box and two rooms)	Box girder (one box and three rooms)
Beam depth	4.5 m (center)	4.5 m (center)
Beam width	16.5 m	20.25 m
Rotating length	46 m + 46 m	46 m + 46 m
Rotating angle	119.06°	120.06°
Rotating weight	5700 t	7200 t

The main technical problems are as follows: Rotation constructions across existing railway lines; Both rotating angles are large (119.06° and 120.06°); Two bridges are rotating at the same time.

In addition, the rotating time must be strictly controlled within the requested skylight time. To ensure structural stability during the rotation, construction control must be strengthened. Because the superstructure is in a dangerous maneuvering state during the rotating process, and the larger the rotating angle, the longer the required rotating time and the longer the bridge is in a dangerous state.

**Figure 2:**
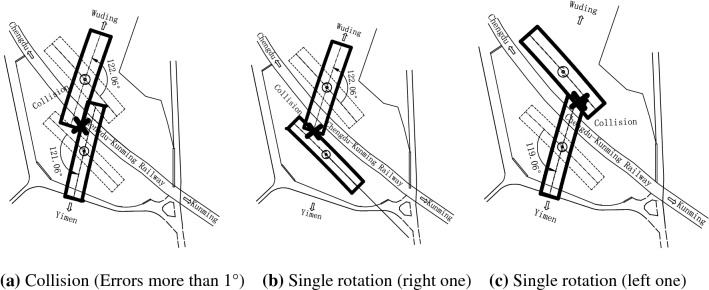
Construction accidents.

After two bridges rotate to their design positions, the distance between them is only 0.5 m. It means that beams collision may occur if the two rotating errors add up to more than 1°, as shown in Fig. 2a. And if bridges are rotated one by one, the position of the first rotating bridge will coincide with the position of another construction after rotating, as shown in Fig. 2b,c. Therefore, it is necessary to carry out a synchronous rotation construction. To meet the above requirements, strict construction control is required for the whole process, such as the rotating speed, the rotating acceleration, angular velocity, and the angular difference between the two bridges.

## Key construction technologies

### Installation process of the spherical hinge

**Figure 3:**
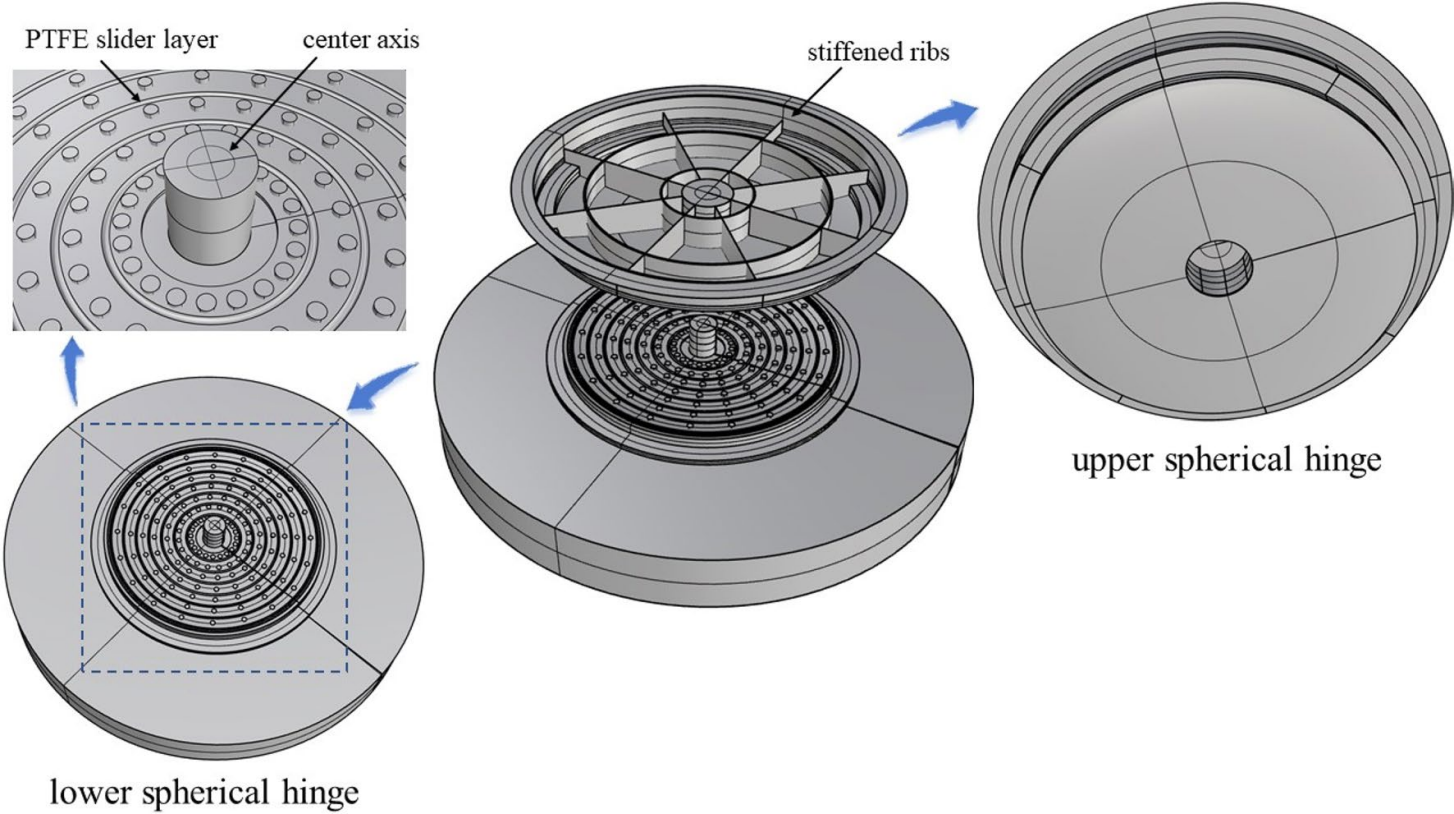
Diagram of spherical hinge structure.

The spherical hinge of these two T-shaped rigid frame bridges is divided into upper and lower turntable systems, which consist of the spherical surface panel, stiffened ribs, center axis and polytetrafluoroethylene (PTFE) slider layer etc. The structure composition of the spherical hinge is shown in Fig. 3.

**Figure 4:**
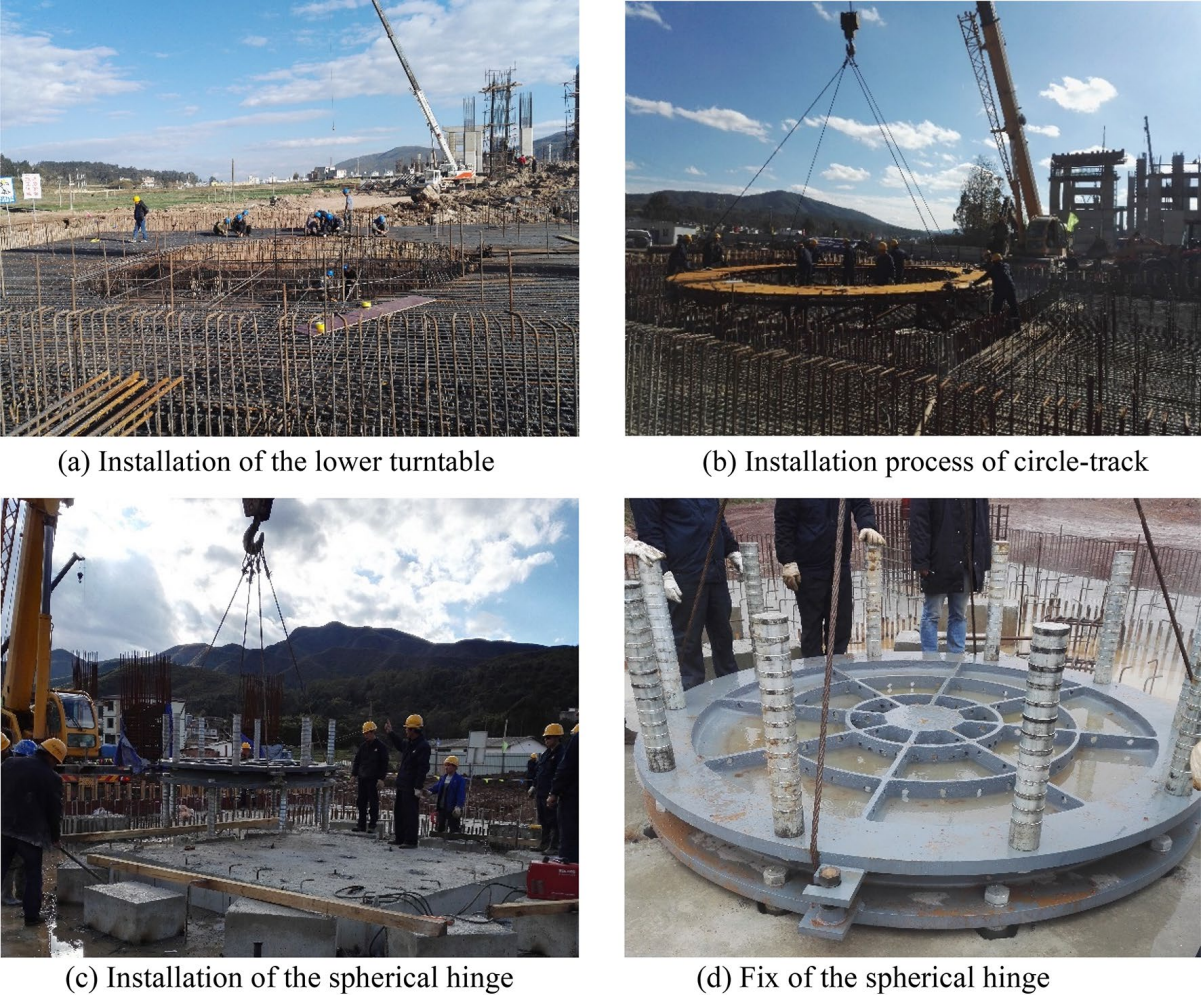
Installation process of the spherical hinge.

The first step of spherical hinge construction is pouring the lower turntable. After that, installation of spherical hinges and circular-track are conducted at the same time. Before installing the PTFE slider, the top surface of the lower spherical hinge is cleaned. The steel spherical hinge used in this project is prefabricated in advance and then transported to the construction site for installation and fixation. Its back is provided with ribs to prevent deformation during processing and transportation. It also facilitates the positioning and strengthening of the connection with the surrounding concrete. After installation of the PTFE slider, butter and PTFE powder should be spread on the spherical surface to evenly fill the space between PTFE sliders. The upper and lower spherical hinges are connected and fixed firmly with bolts. The upper spherical hinge is placed on the lower one by aligning with the center axis. After cleaning up the butter that has been squeezed out, the gap between the edges of the upper and lower spherical hinges should be sealed by wide tape. The process of the installation of the spherical hinge is illustrated as shown in Fig. 4. PTFE sliders are in a high compressive stress state, and the average calculated compressive stress is 57.7 MPa, smaller than its. design compressive strength 100 MPa.

The core of the entire rotating support member is a spherical hinge, and the accuracy of its installation and the density of the concrete underneath are the key factors affecting the success or failure of the swivel. In order to ensure the installation quality of the ball hinge, the ball hinge bracket is generally preset in the turntable, and the screw head on the bracket is used to accurately positioning the integrally suspended ball hinge. At the same time, to ensure the compactness of the concrete under the ball hinge, a vibrating hole (also a vent hole) is opened in the ball hinge before pouring to ensure the vibrating quality of the concrete. As concrete, it is guaranteed from the mix proportion and fluidity, and the micro-expansion concrete with good fluidity is used.

### Design of traction system

As the key point of rotation construction, the traction system determines whether the rotation construction can be completed safely and rapidly or not. When rotation starts, the traction force must overcome the static friction between the upper and lower spherical hinge, the static friction between the circular track and support columns. During the rotation process, the traction force will be changed to overcome the dynamic friction. The traction jacks are symmetrical arranged along with the turntable and are tangent to the outer profile of the turntable. Two bunches of traction cables have been set on the upper turntable along with the given tracks through the jacks after twining around for a 3/4 circle. Traction jacks on both sides provide a set of horizontal rotating couples, which pull the steel strands anchored and wound on the turntable to make the superstructure rotate. It’s extremely important for the rotational smoothness and kinematical control of superstructure during the rotation, which has been studied by many scholars^24–30^.

In addition to the traction system, a booster system, a fine-tuning system, and a limit system are also used to ensure the accuracy of rotation. The booster system is used to overcome the difference between static and dynamic friction moments and to start the entire rotation. The Booster system is composed of a booster distribution beam and booster jacks, which is installed between steel support columns and booster reaction frame. The fine-tuning system is to adjust possible deviations in time. Four YCW6000 jacks are set symmetrically under the turntable to adjust the posture of the superstructure in longitudinal and transverse directions before sealing the turntable. The Limit system is to ensure that beam does not continue moving after rotating in place, and it works by installing booster jacks and booster reaction frame in reverse before rotating in place.

### Precision control method

During the construction process, in order to make the alignment of bridge and stress state consistent with the design, a scientific and reasonable construction control method is essential. For the T-type rigid frame bridge constructed by the rotating method, multiple conversion of the stress system makes construction control more important. For the safety and stability of the structure, the sectional stress of the member must below the material strength during the construction^31–33^, therefore, the stress of bridge needs to be monitored by the sensor.

#### Stress measured points arrangement of sub-turntable

**Figure 5:**
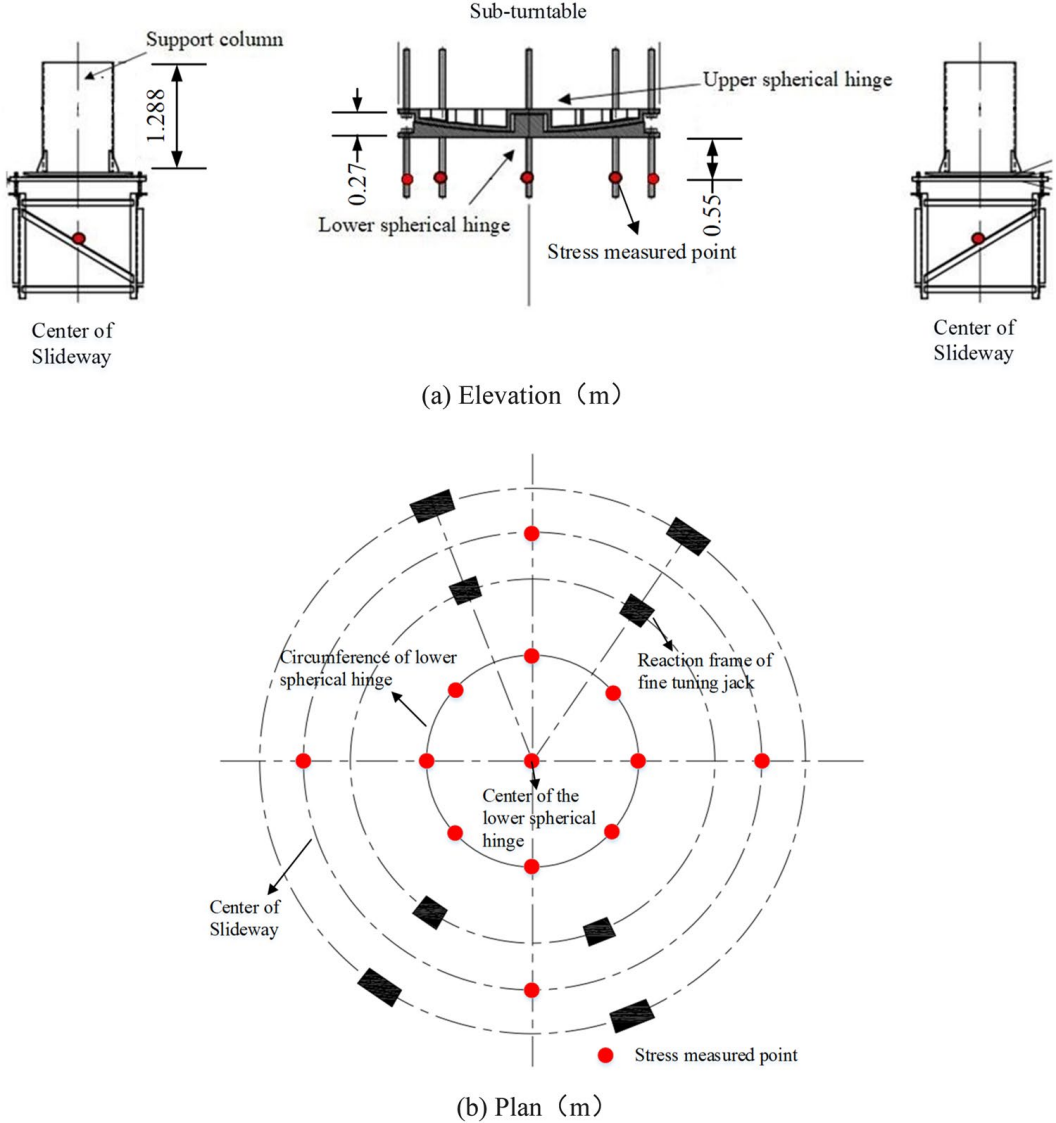
Stress measured point arrangement of sub-turntable.

A total of 13 observation points are distributed on the sub-turntable, including one below the center of the lower spherical hinge, eight around the circumference of it, and four under the circular track. A layout of the arrangement is shown in Fig. 5. The purpose is to obtain information about stress changes of the sub-turntable. When support sandbox and temporary fixation are removed, the spherical hinge will shift due to eccentricity. The attitude adjustment of the rotating structure can be targeted, according to the stress changes of the sub-turntable. During the rotating process, the stress changes of the lower spherical hinge and circular track can directly reflect the stability. By monitoring it, the stress state of the rotating structure can be obtained in real-time, and emergency measures can be taken for the emergent situation.

#### Stress measured points arrangement of support columns

**Figure 6:**
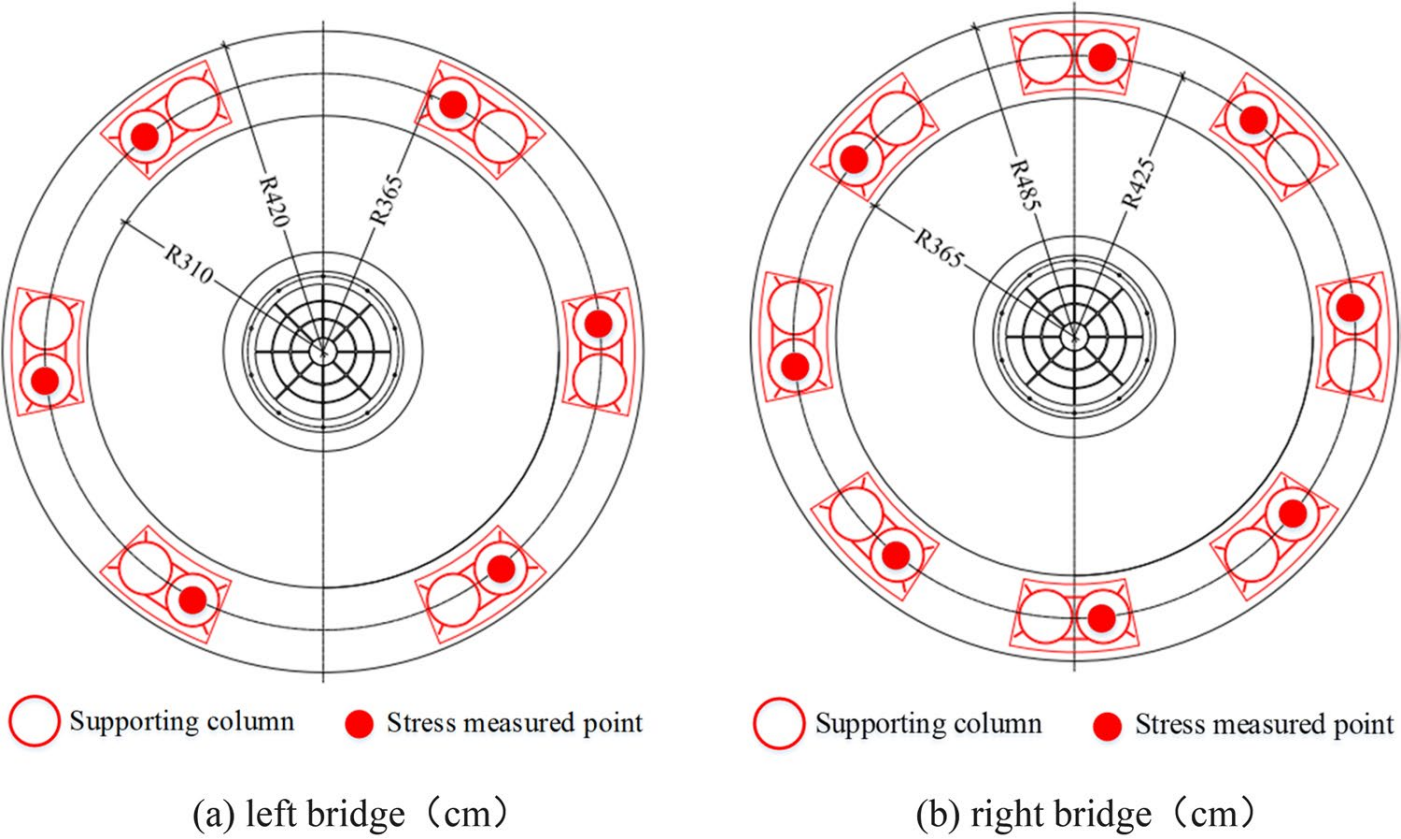
Stress measured points arrangement of supporting columns.

The method of monitoring the stress of supporting columns is to embed the strain gauge in each one of them. The left bridge has 6 measuring points and the right one has 8 measuring points, which is shown in Fig. 6. When support columns are in contact with the circular track, the stress of the circular track changes, which reflects the eccentricity of the rotation structure and provides a theoretical basis for the adjustment of the center of gravity.

#### Stress measured point arrangement of the main pier

The main pier is arranged with 4 measured points at a height of 2.0 m from the top surface of the pile cap. By monitoring the stress difference on both sides of the main pier, the eccentric moment of the rotating structure can be calculated, thus the counterweight can be arranged as well. The stress changes of the main pier can also directly reflect the stability of the structure during the rotating process.

#### Stress measured point arrangement of box girder

**Figure 7:**
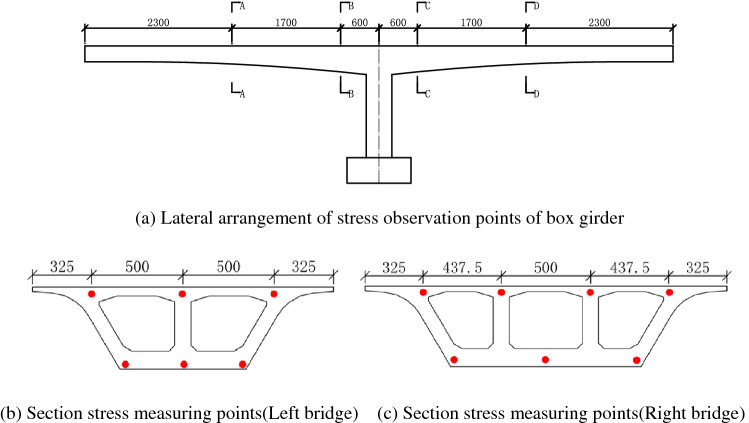
Arrangement of stress measured of girder (cm).

The lateral arrangement of stress observation points of the box girder is shown in Fig. 7a.The sections of the box girder for stress measurements are distributed at the middle and end of the cantilever, as well as both sides of the T-shape rigid, which are shown in Fig. 7b,c.

The left bridge has 6 strain sensors on each observation section while the right bridge has 7 ones. It should be ensured that the distance between the embedded position and the anchorage end of the prestressed steel beam is greater than 1.5 m so that the results of the sensor are not affected by the stress concentration of the prestressed concrete’s anchorage end.

## Theoretical analysis of key parameters

### Unbalanced moment

The unbalanced moment (also known as the overturning torque^34^) of the rotating structure includes the unbalanced moment generated by the structure itself and unbalanced that generated during the construction process. The former can be calculated by finite element analysis which has been widely studied^35–38^, and the latter needs to be tested on the construction site.

#### Finite element model and lateral unbalanced moment

A full-scale bridge was modeled by Midas/Civil and ANSYS separately, The Midas/Civil model uses beam elements to simulate. In the ANSYS model, the concrete is simulated by the solid 45 element, and the prestressed tendon is simulated by the link 8 element. The 3D beam element in Midas/Civil is “fake 3D”, it is actually a planar bar model. Solid 45 element is used for three-dimensional solid structure, and the element is defined by 8 nodes. Each node of the element has three degrees of freedom along the X, Y, Z axis. Link 8 element can only be used for single-axis tension and compression bar element.

The bottom of the pier is fixed during the calculation of the superstructure of the bridge. The connection type between the pier top and beam adopts the rigid connection, and its connection stiffness is 10^6^ times the maximum stiffness of the entire model. Beam end supports only constrain the vertical direction and release all other directions. In the simulation of the construction stage, the full space support construction is simulated by the pressure-bearing bearing only. And support stiffness is empirically between 10^6^ and 10^7^ kN/m. The support stiffness of these two models is 5 × 10^6^ kN/m.

The results of the two models are summarized in Table 2.

**Table 2:** Lateral unbalanced moment of two models.

	Theoretical value of Midas/Civil (kN m)	Theoretical value of ANSYS (kN m)	Difference (%)
Left bridge	7585	7190	5.2
Right bridge	9894	9085	8.2

Difference = (D_Midas_ − D_ANSYS_)/D_Midas_.

From the above analysis of the lateral unbalanced moment, it can be found that the calculation results of the ANSYS are smaller than those of the Midas/Civil. The reason is that when Midas/Civil uses the beam element to simulate, its node position is on the centerline of the beam, which means the center of gravity of the beam element is also on the centerline of the beam. In fact, the curve bridge’s center of gravity is located on the outside of the centerline of the beam, so it causes the simulated force arm larger than the actual situation. Eventually, the Midas/Civil calculation result of the lateral unbalanced moment is relatively larger.

**Figure 8:**
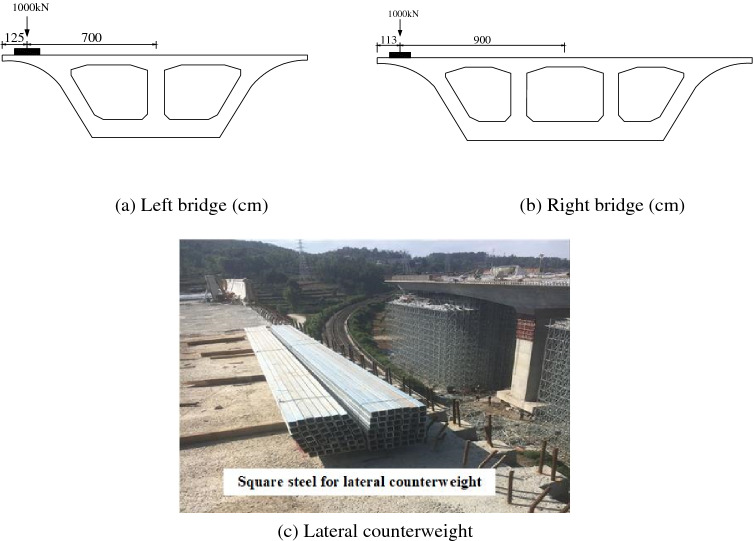
Left and right bridge’s lateral counterweight.

According to the actual situation of the site, the square steel is adopted for lateral counterweight. Both of the counterweights are 1000 kN, and the counterweight moments are 7000 kN m and 9000 kN m, respectively. The lateral counterweights of the left and right bridge are shown in Fig. 8. It is worth noting that lateral counterweight needs to be set before the removal of support or before the removal of the sandbox. When determining the lateral counterweight scheme, the lateral frame calculation of the flange plate should be carried out to ensure that the force state meets the requirements.

#### Test of longitudinal unbalanced moment

Through the previous research on the unbalanced moment test of the rotating structure, the main test scheme of longitudinal unbalanced moment of the rotating structure is called the spherical hinge rotation method.

The spherical hinge rotation method regards the rotation of structure around the spherical hinge as a rigid body rotation, and the force balance equation is established by the rotation of the rigid body from static friction to dynamic friction.

The test processes are as follows: several jacks are symmetrically arranged around the spherical hinge for jacking the spherical hinge; the jacking force and displacement are recorded by dial gauge and force sensor; then the force–displacement curve is plotted based on the data obtained. When the spherical hinge produces a significant displacement during jacking or falling, the reading of the force sensor should be recorded. Then the reading is entered into the established force balance equation which can be used to calculate the spherical hinge friction moment *M*_*Z*_ and the unbalanced moment *M*_*G*_.

The number of jacks on one side of the structure can be one or two, depending on the rotating body and working space. Based on the magnitude of the unbalanced force of the rotating body, there may be two actual situations.

(1) Spherical hinge friction moment *M*_*Z*_ is greater than unbalanced moment *M*_*G*_*.*

**Figure 9:**
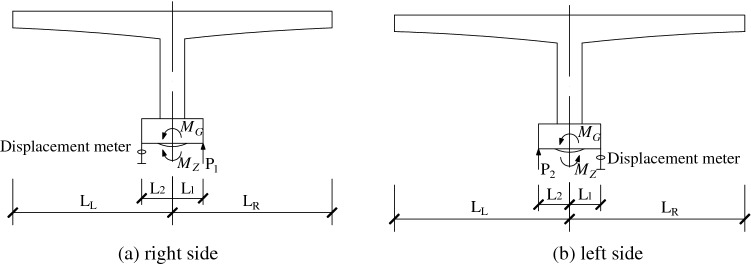
Equilibrium state of rotating structure^21^.

When the sandbox and temporary consolidation of the spherical hinge are removed, it can be judged that the spherical hinge friction moment is greater than the unbalanced moment if the beam body doesn’t rotate around the spherical hinge. Then the jacks are applied on both sides to jack the structure above the spherical hinge and changes in jacking force and displacement are recorded, as shown in Fig. 9a.

When the displacement changes significantly, the magnitudes of jacking force on both sides are recorded as *P*_1_ and *P*_2_. The following balance equations can be obtained from the above two different sides of jacking forces^21^.

1 $$P_{1} L_{1} + M_{{\rm G}} = M_{{\rm Z}}$$

2 $$P_{2} L_{2} = M_{{\rm G}} + M_{{\rm Z}}$$

Then the unbalanced moment *M*_*G*_ and the frictional moment *M*_*Z*_^21^ can be obtained.

3 $$M_{{\rm G}} = \frac{{P_{2} L_{2} - P_{1} L_{1} }}{2}$$

4 $$M_{{\rm Z}} = \frac{{P_{1} L_{1} + P_{2} L_{2} }}{2}$$

(2) Spherical hinge friction moment *M*_*Z*_ is less than unbalanced moment *M*_*G.*_

When the sandbox and temporary consolidation of the spherical hinge are removed, it can be judged that the left supporting column is in contact with slideway if the rotating structure’s center of gravity is biased to the left. The rotating structure is jacked on the left side, as shown in Fig. 9b. When the displacement changes significantly, the magnitude of jacking force is recorded as ${P}_{2}$. Then we let the jack fall off and unload, the magnitude of jacking force is record as ${P}_{2}{^{\prime}}$ when the system slips in the opposite direction.

According to the equilibrium equations in the two cases, the unbalanced moment and the friction moment are also obtained^21^.

5 $$M_{{\rm G}} = \frac{{P_{2} L_{2} + P^{\prime}_{2} L_{2} }}{2}$$

6 $$M_{{\rm Z}} = \frac{{P_{2} L_{2} - P^{\prime}_{2} L_{2} }}{2}$$

7 $$e_{0} = \frac{{M_{{\rm G}} }}{N}$$

$M_{{\rm Z}}$—Friction moment of spherical hinge; $M_{{\rm G}}$—Rotating body imbalance moment; ${\text{N}}$—Rotating weight; $e_{0}$—Front body eccentricity.

The spherical hinge rotation method is clear and it only involves the force balance equation of the rotating structure. If the jacking force can be accurately tested at the moment that the spherical hinge starts rotating, a more accurate unbalanced moment value can be obtained.

In addition, this method can not only measure the unbalanced moment of the rotating structure but also measure the frictional moment. Finally, the values of the key parameters of the rotating system, such as friction coefficient, eccentricity, and rotating traction force can be calculated. This method is generally applied to test the longitudinal unbalanced moment, however, the transversal one is difficult to measure due to the limitation of the construction space, and the time cost of this method is high.

(3) Force–displacement curve (P–L curve).

**Figure 10:**
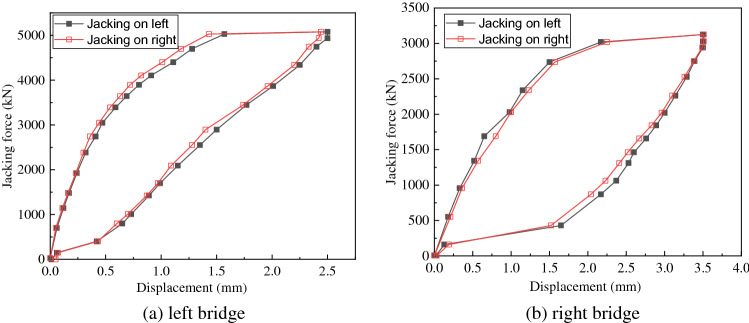
P–L curve.

The load–displacement test results of the bridge are plotted as a load–displacement curve (the P–L curve). When the displacement increases rapidly and the load decreases, it indicated that the spherical hinge has already rotated. The value of the jacking force is determined according to the critical state when the spherical hinge is about to rotate. Before weighing, the supporting columns of the left and right bridge are in contact with the slideway. So we first judge that the spherical hinge friction moment *M*_*Z*_ is smaller than the longitudinal unbalanced moment *M*_*G*_. P–L curves of the left and right bridge are shown in Fig. 10.

It can be seen from Fig. 10a that when the jacking force reaches 5079 kN, the displacement of the left bridge’s rotating structure increases rapidly from 1.6 to 2.6 mm. When the jacking force drops to 150 kN, the displacement decreases rapidly from 0.42 to 0.06 mm. Thus, it can be judged that the jacking forces of the left bridge $P_{2}$ and $P_{2}{^{\prime}}$ are 5079 kN and 150 kN, respectively.

It can be seen from Fig. 10b that when the jacking force reaches 3020 kN, the displacement of the right bridge’s rotating structure increases rapidly from 2.2 to 3.5 mm. When the jacking force drops to 155 kN, the displacement decreases rapidly from 1.65 to 0.13 mm. Thus, it can be judged that the jacking forces the right bridge $P_{2}$ and $P_{2}{^{\prime}}$ are 3020 kN and 155 kN, respectively.

### Determination of the static friction coefficient

**Figure 11:**
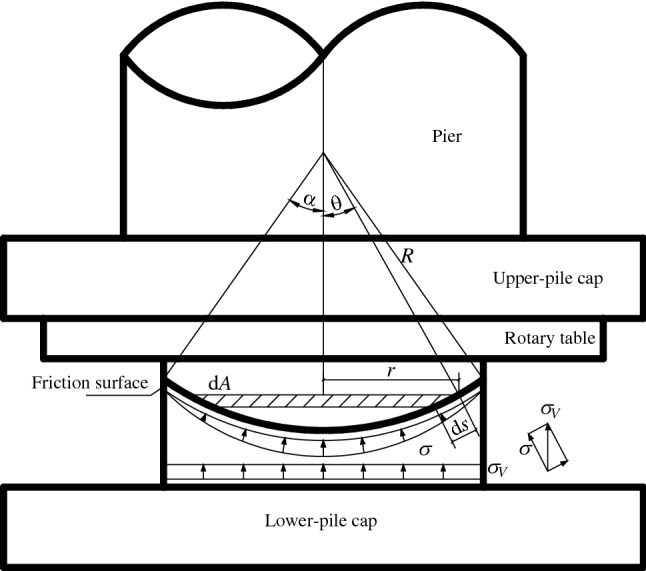
Calculating diagram of spherical hinge’s static friction coefficient.

The normal method for calculating the static friction coefficient of the spherical hinge is to perform the weighing experiment. When the spherical hinge rotates slightly in the vertical plane of the rotating structure, the friction moment is the sum of the moments of each micro-area on the friction surface to the normal of the center of the spherical hinge. Calculation assumptions include the uniformly distributed pressure on the spherical hinge, a contactless between the supporting columns and the slideway, and ignoring the influence of wind load and temperature on the static friction coefficient. Under these conditions, the static friction coefficient calculation model of the spherical hinge is shown in Fig. 11.

It is assumed that

$${\rm d}M_{{\rm Z}} = R\cos \theta {\rm d}F;$$

The values of items in the formula are as follow:

$${\text{d}}F = \mu_{0} \sigma {\text{d}}A,\quad {\text{d}}A = 2\pi rds,\quad r = R\sin \theta ,\quad {\text{d}}s = R{\text{d}}\theta ,\quad \sigma { = }\sigma_{vert} \cos \theta ,$$

$$\sigma_{vert} = \frac{N}{{\pi R^{2} \sin^{2} \alpha }};$$

$${\text{d}}M_{{\rm Z}} = \frac{{2\mu_{0} NR\sin \theta \cos^{2} \theta }}{{\sin^{2} \alpha }}d\theta {;}$$

With integration, we can get the following formula:

8 $$M_{{\rm Z}} { = }\int_{0}^{\alpha } {{\text{d}}M_{{\rm Z}} = \frac{{2(1 - \cos^{3} \alpha )}}{{3\sin^{2} \alpha }}\mu_{0} NR}$$

Then the spherical hinge static friction coefficient is as follows:

9 $$\mu_{0} { = }\frac{{3\sin^{2} \alpha }}{{2(1 - \cos^{3} \alpha )}}\frac{{M_{{\rm Z}} }}{NR}$$

$M_{{\rm Z}}$—Friction moment of the spherical hinge; ${\mu }_{0}$—Spherical hinge’s static friction coefficient; ${\text{N}}$—Rotating structure’s gravity; ${\text{R}}$—Radius of spherical hinge’s rotary table.

The above static friction coefficient derivation process assumes that the friction moment is the sum of the moments of each micro-area on the friction surface to the normal of the center of the spherical hinge.

The distance from each position on the micro-ring to the axis of rotation is different. In the formula ${\rm d}M_{{\rm Z}} = R\cos \theta {\rm d}F$, the middle force arm ($R\cos \theta$) is the distance from the micro-ring to the normal line of the spherical hinge. That leads to a certain error during the calculation of the static friction coefficient by using the above method. Furthermore, it may results in a large error in the rotating traction force compared with the measured result on site.

**Figure 12:**
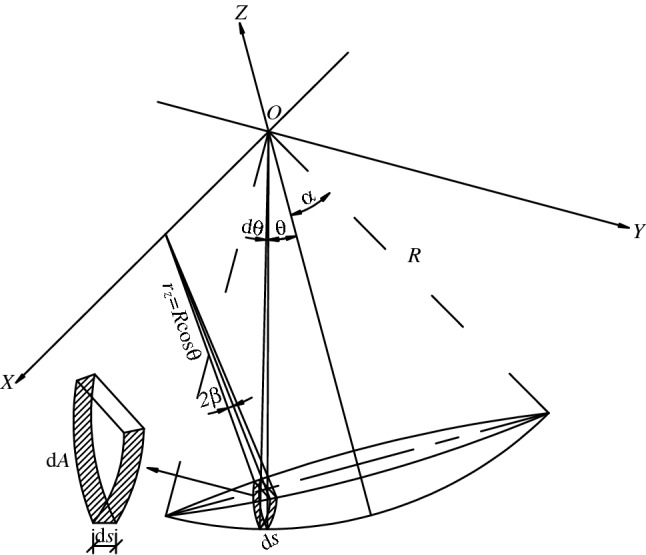
Modified calculating diagram of spherical hinge’s static friction coefficient.

In order to calculate the spherical hinge static friction coefficient accurately, Yan^22^ modified the above formula, and the modified calculating diagram is shown in Fig. 12. When performing longitudinal unbalanced weighing, we assume that the spherical hinge rotates a micro-angle around the OX axis and the spherical hinge is divided into countless micro-elements along the OX axis. Then we can find that the radius of every micro-element is $r_{z} = R\cos \theta$, which means the distance from each micro-element to the OX axis is $r_{z}$, where the parameter of the spherical hinge $\alpha$ is half of the sphere center angle of the spherical hinge.

Namely,

$${\text{d}}M_{z} = r_{z} {\text{d}}F,\quad {\text{d}}F = \mu_{0} \sigma {\text{d}}A,\quad {\text{d}}A = 2\beta r_{z} {\text{d}}s,\quad \sigma { = }\frac{N}{{\pi R^{2} \sin^{{2}} \alpha }},$$

We can find that ${\text{d}}M_{{\rm Z}} = \frac{{2\mu_{0} NR(\alpha - \theta )\cos^{2} \theta }}{{\pi \sin^{2} \alpha }}{\rm d}\theta {;}$

10 $$M_{{\rm Z}} { = 2}\int_{0}^{\alpha } {{\text{d}}M_{{\rm Z}} = \frac{{4\left( {\frac{1}{8}{ + }\frac{{\alpha^{2} }}{4} - \frac{\cos 2\alpha }{8}} \right)}}{{\pi \sin^{2} \alpha }}\mu_{0} NR}$$

And the static friction coefficient of spherical hinge is

11 $$\mu_{0} = \frac{{\pi \sin^{2} \alpha }}{{4\left( {\frac{1}{8}{ + }\frac{{\alpha^{2} }}{4} - \frac{\cos 2\alpha }{8}} \right)}}\frac{{M_{{\rm Z}} }}{NR}$$

It is worthy to mention that, $\sigma$ ($\sigma = \frac{N}{{\pi R^{2} \sin^{2} \alpha }}$) used in the study^22^ is vertical stress. Nevertherless here $\sigma$ is radial stress and its accurate value is $\sigma = \frac{N\cos \theta }{{\pi R^{2} \sin^{2} \alpha }}$.

And we can find that ${\text{d}}M_{{\rm Z}} = \frac{{2\mu_{0} NR(\alpha - \theta )\cos^{3} \theta }}{{\pi \sin^{2} \alpha }}{\rm d}\theta {;}$

12 $$M_{{\rm Z}} { = 2}\int_{0}^{\alpha } {{\text{d}}M_{{\rm Z}} = \frac{{4\left( {\frac{7}{9} - \frac{2\cos \alpha }{3} - \frac{{\cos^{3} \alpha }}{9}} \right)}}{{\pi \sin^{2} \alpha }}\mu_{0} NR}$$

And the static friction coefficient of the spherical hinge is

13 $$\mu_{0} = \frac{{\pi \sin^{2} \alpha }}{{4\left( {\frac{7}{9} - \frac{2\cos \alpha }{3} - \frac{{\cos^{3} \alpha }}{9}} \right)}}\frac{{M_{{\rm Z}} }}{NR}$$

In the above derivation, the contact surface of the spherical hinge is simplified into a plane contact. It is considered that the plane of the spherical hinge is affected by the uniform force. However, it is actually a curved surface contact, and the stress distribution is complicated. Therefore, the spherical hinge static friction coefficient should be further corrected.

For concentrated forces acting on a hemispherical body, its radial stress can be solved by elastic mechanics. Radial stress expression is as follow:

14 $$\sigma_{{\rm p}} = - \frac{2F}{\pi }\frac{\cos \theta }{\rho }$$

$\sigma_{{\rm p}}$—Radial stress; $F$—Concentrated forces acting on a hemispherical body; $\theta$—The angle between a line (between a point on a sphere and its center) and the normal to the sphere; $\rho$—Ball radius.

The stress of the spherical hinge can be calculated by referring to the hemispherical radial stress expression. The stress expression of the spherical hinge can be written as

15 $$\sigma = f(R,N,\alpha )\cos \theta$$

Because $$N = \int {\sigma {\rm d}A = \int_{0}^{\alpha } {2\pi R^{2} \sin \theta (f\cos^{2} \theta ){\rm d}\theta = \frac{{2\pi fR^{2} }}{3}} } (1 - \cos^{3} \alpha )$$.

We can find that

16 $$f = \frac{3N}{{2\pi R^{2} (1 - \cos^{3} \alpha )}}$$

It can be substituted into the formula (15)^39^

17 $$\sigma = \frac{3N\cos \theta }{{2\pi R^{2} (1 - \cos^{3} \alpha )}}$$

We can find that

18 $$M_{{\rm Z}} = 2\int_{0}^{\alpha } {{\rm d}M_{{\rm Z}} = \frac{{3\left( {\frac{7}{9} - \frac{2\cos \alpha }{3} - \frac{{\cos^{3} \alpha }}{9}} \right)}}{{\pi (1 - \cos^{3} \alpha )}}} \mu_{0} NR$$

And the static friction coefficient of the spherical hinge is

19 $$\mu_{0} = \frac{{\pi (1 - \cos^{3} \alpha )}}{{3\left( {\frac{7}{9} - \frac{2\cos \alpha }{3} - \frac{{\cos^{3} \alpha }}{9}} \right)}}\frac{{M_{{\rm Z}} }}{NR}$$

### Counterweight

According to the results of jacking force measured by the weighing experiment, the values of the spherical hinge friction moment, the longitudinal unbalanced moment, the spherical hinge static friction coefficient can be calculated by the theory mentioned in the previous section.

According to the preliminary judgment, the spherical hinge friction moment is smaller than the longitudinal unbalanced moment of the rotating structure, hence the spherical hinge friction moment can be calculated by the formula (3), the longitudinal unbalanced moment of the rotating structure can be calculated by the formula (4), and the eccentricity $e_{0}$ is calculated according to formula (20).

20 $$e_{0} = \frac{{M_{Z} }}{N}$$

After weighing, the longitudinal unbalanced moment of the left bridge is calculated to be 9771 kN m, and the eccentricity is 17.2 cm; the longitudinal unbalanced moment of the right bridge is 6542 kN m, and the eccentricity is 9.2 cm.

The rotating traction force $T$ is calculated according to the formula (21). The static friction coefficient of the spherical hinge is calculated according to the modified formula (19). The calculated value of the rotating traction force of the left bridge is 176 kN and the right one is 110 kN.

21 $$T = \frac{{2\mu_{{0}} RN}}{3D}$$

**Figure 13:**
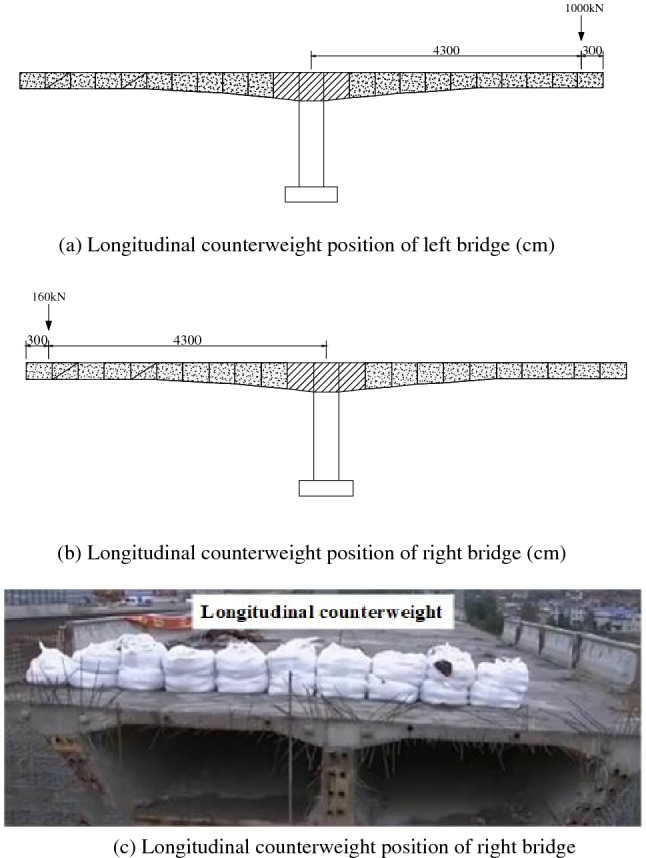
Longitudinal counterweight.

The longitudinal unbalanced moment and eccentricity of the left bridge are large, so it is proposed to adopt the longitudinal tilting counterweight scheme. The proposed counterweight position is 3 m from the right end of the cantilever and the proposed counterweight is 160 kN. The longitudinal unbalanced moment and eccentricity of the right bridge are relatively small, so it is proposed to adopt the balanced counterweight scheme. The proposed counterweight position is 3 m from the right end of the cantilever and the proposed counterweight is 160 kN (Fig. 13).

The eccentricity after counterweight can be calculated according to the following formula:

22 $$e^{\prime} = \frac{{(Ne_{0} - W^{\prime}l^{\prime})}}{N + W^{\prime}}$$

$e^{\prime}$—Eccentricity after counterweight; $W^{\prime}$—Counterweight; $l^{\prime}$—Distance from counterweight position to the centerline.

After the longitudinal counterweight, the total weight of the left bridge’s rotating structure is 56,900 kN, and the right one is 71,200 kN. Based on the calculation data in Table 3, the static friction coefficients of the left and right bridge’s spherical hinge calculated by weighing experiments are 0.044 and 0.02, respectively. However, to prevent the inability to rotate due to insufficient estimation, the static friction coefficient of 0.1 and the dynamic friction coefficient of 0.06 are used for the selection of the traction device. The static friction coefficient of the spherical hinge calculated by the weighing experiment is used to calculate the traction force’s expectation value to guide the on-site rotating construction.

**Table 3:** Statistical table of key parameters’ calculation data.

Key parameters	Left bridge	Right bridge
Rotating weight $N$ (kN)	56,740	71,040
Spherical radius of rotary table $R$ (m)	5.94	6.74
Jacking force $P_{{1}}$ (kN)	5079	3020
Jacking force $P_{2}$ (kN)	150	155
Spherical hinge friction moment $M_{Z}$ (${\text{kN}} \cdot {\text{m}}$)	9210	5912
Unbalanced moment $M_{G}$ (${\text{kN}} \cdot {\text{m}}$)	9771	6552
Eccentricity $e_{0}$ (cm)	17.2	9.2
Static friction coefficient $\mu_{0}$	0.044	0.020
Traction force $T$ (kN)	176	110
Eccentricity after counterweight (cm)	5.1	− 0.5

Both the left and right bridges use two sets of LSD200 systems with hydraulic, synchronous, and automatic continuous traction to form the horizontal rotating couples. The 15-ΦS15.2 steel strands are anchored and wound around the circumference of the 850 (950) cm turret. The traction system rotates the rotating system by pulling the steel strands.

## Discussion of results

### The control strategy of SRM

Two parts of the bridge were rotated simultaneously during construction, and the design rotation angle of the left one is 119.06° while the right one is 120.06°. Considering the stability and safety of construction, the design angular velocity w is smaller than 1.15°/min. The construction process was divided into three stages. The first stage was trial tuning stage, the left bridge rotated 5° counterclockwise, and the right bridge rotated 6° counterclockwise, the left and right bridge must be adjusted to the same angle. During the trial operation, the inertial braking distance was tested. The second was the formal turning stage, which started from 114.06° of two bridge, stopped after rotating 111° with a uniform speed of 1.15°/min. The third was the fine-tuning stage with the speed of 0.3°/min, which finalized the last 3.06° with four turns. The first three turns, the bridge rotated 1° each turn, and the last turn, the bridge rotated 0.06°.

The distance between the left and right bridge is only 50 cm when rotation finished. Therefore, the rotation velocity and angle amplitude need to be strictly controlled during the execution of synchronous rotation construction of bridge. The rotation velocity should be controlled within 2°/min at the trial operation and formal turning stages, and within 0.2°/min at the fine-tuning stage. these measures can effectively ensure the safety and stability of the bridge in the rotation process, and avoid the collision of two bridges.

### The calculation formula of static friction coefficient

The characteristics of static friction coefficient of each formula are shown in Table 4. After substituting the spherical hinge parameters of the project in this project into the above formulas, the calculation results of the static friction coefficient are shown in Table 5. The calculation results show that the calculation results according to the traditional formula (9) differ greatly from the calculation results according to the modified formula (11), formula (13), and formula (19). The value of the static friction coefficient calculated by the traditional method is too small resulting in a large difference between the calculated value and the actual value of the rotating traction force. The difference between the calculation results of formula (11), formula (13), and formula (19) are small. The formula (19) is the most accurate theoretical derivation. This study will verify the accuracy of the calculated traction force by comparing it with the measured one.

**Table 4:** Characteristics of static friction coefficient of each formula.

Formula	Static friction coefficient coefficient	Characteristics
Formula (9)	$\mu_{0} { = }\frac{{3\sin^{2} \alpha }}{{2(1 - \cos^{3} \alpha )}}\frac{{M_{{\rm Z}} }}{NR}$	Traditional calculation formula. Micro-element’s friction moment is ${\rm d}M_{{\rm Z}} = R\cos \theta {\rm d}F$, The microelement’s arm of force is the distance from each point to the center of the spherical hinge $R\cos \theta$
Formula (11)	$\mu_{0} = \frac{{\pi \sin^{2} \alpha }}{{4\left( {\frac{1}{8}{ + }\frac{{\alpha^{2} }}{4} - \frac{\cos 2\alpha }{8}} \right)}}\frac{{M_{{\rm Z}} }}{NR}$	The literature^22^ corrected calculation formula. Micro-element’s friction moment is ${\rm d}M_{{\rm Z}} = r_{{\rm Z}} {\rm d}F$. The microelement’s arm of force is the distance from each point to the center of the spherical hinge $r_{{\rm Z}}$. The spherical hinge’s stress is simplified to the plane contact stress $\sigma = \frac{N}{{\pi R^{2} \sin^{2} \alpha }}$, This stress is vertical stress rather than radial stress
Formula (13)	$\mu_{0} = \frac{{\pi \sin^{2} \alpha }}{{4\left( {\frac{7}{9} - \frac{2\cos \alpha }{3} - \frac{{\cos^{3} \alpha }}{9}} \right)}}\frac{{M_{{\rm Z}} }}{NR}$	Correct the formula deduced by literature^22^*,* and the stress of the spherical hinge takes radial stress $\sigma = \frac{N\cos \theta }{{\pi R^{2} \sin^{2} \alpha }}$
Formula (19)	$\mu_{0} = \frac{{\pi (1 - \cos^{3} \alpha )}}{{3\left( {\frac{7}{9} - \frac{2\cos \alpha }{3} - \frac{{\cos^{3} \alpha }}{9}} \right)}}\frac{{M_{{\rm Z}} }}{NR}$	Correct the formula deduced by literature^22^, and the stress of the spherical hinge takes the method of elastic mechanics. And outcome is $\sigma = \frac{3N\cos \theta }{{2\pi R^{2} (1 - \cos^{3} \alpha )}}$

**Table 5:** Spherical hinge’s static friction coefficient.

Position	Spherical radius *R* (m)	Plane radius r (m)	Spherical hinge parameter *α* (°)	Formula (9)	Formula (11)	Formula (13)	Formula (19)
Left one	5.94	0.9	17.43	$\frac{{M_{{\rm Z}} }}{0.977NR}$	$\frac{{M_{{\rm Z}} }}{0.647NR}$	$\frac{{M_{{\rm Z}} }}{0.642NR}$	$\frac{{M_{{\rm Z}} }}{0.627NR}$
Right one	6.74	1.1	18.79	$\frac{{M_{{\rm Z}} }}{0.975NR}$	$\frac{{M_{{\rm Z}} }}{0.648NR}$	$\frac{{M_{{\rm Z}} }}{0.643NR}$	$\frac{{M_{{\rm Z}} }}{0.626NR}$

**Figure 14:**
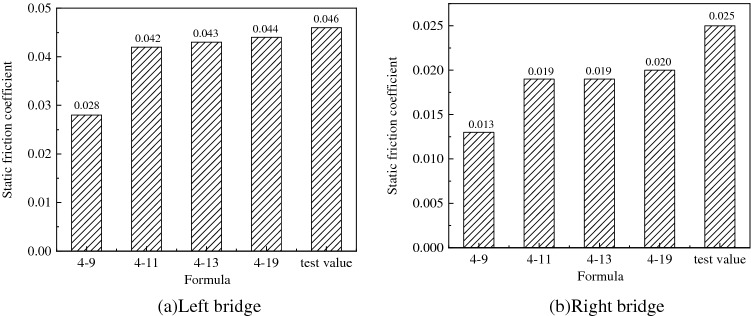
Comparison of calculated values of different formulas.

The starting traction force of the left and right bridges are 193 kN and 137 kN, respectively. The actual static friction coefficients of the left and right spherical hinges calculated by the actual traction forces are 0.046 and 0.025, respectively, which are consistent with the calculations of 0.044 and 0.02 according to formula (19). Results of different formulas are compared in Fig. 14. The accuracy of the formula is verified through these two bridge constructions. It is recommended to calculate the static friction coefficient of the spherical hinge according to the formula (19).

## Conclusion

The SRM has been mainly used for the straight-line bridge and in this paper, this method has been extended for curved rigid frame bridges. Taking two bridges of the Wuyi Expressway over the Chengdu-Kunming Railway as an illustration, the construction technology and the key parameters of synchronous rotation execution with large angle T-shape curved rigid frame bridge have been studied. The applicability of SRM in curved bridge construction has been verified. The main conclusions are as followings:

1. The key technologies of T-shaped curved rigid frame bridge utilizing SRM has been introduced, including the construction of ball joint, the design of traction system and monitoring scheme. During the construction of synchronous rotation process of bridge, the rotation velocity and angle amplitude need to be strictly controlled. The rotation velocity should be controlled within 2°/min at the trial operation and formal turning stages, and within 0.2°/min at the fine-tuning stage. With aforementioned measures, the safety and stability of construction process can be guaranteed, and the collision of two components of bridges can be prevented.
2. The weighing experiment has been carried out using the ball joint rotation method for the curved continuous rigid frame, and the jacking force value was obtained by the load–displacement curve. Then, the longitudinal unbalanced moment of the curved rigid frame has been calculated, which provided a basis for predicting the ball joint friction coefficient and swivel construction traction force. It shows that the construction parameters obtained by the spherical joint rotation method are accurate and reliable, according to the smoothness of construction process and Motion posture of the bridge. it proves that the method used in present bridge can be used for weighing experiment of curved rigid frame.
3. The applicability of the existing friction coefficient formula of ball joint in literature has been studied. The static friction coefficient of the ball joint was measured indirectly by the traction force from the field test data of bridge construction. The tested friction coefficient has been compared with analytical ones, and the results show that, the formula proposed by Yan is in good agreement with the actual test results. Therefore it is suggested that Yan formula should be used for the calculation of the static friction coefficient, which will be further unitized for the determination of the traction force.
